# Inhibition of myeloid differentiation primary response protein 88 provides neuroprotection in early brain injury following experimental subarachnoid hemorrhage

**DOI:** 10.1038/s41598-017-16124-8

**Published:** 2017-11-17

**Authors:** Huiying Yan, Dingding Zhang, Yongxiang Wei, Hongbin Ni, Weibang Liang, Huasheng Zhang, Shuangying Hao, Wei Jin, Kuanyu Li, Chun-Hua Hang

**Affiliations:** 1 Department of Neurosurgery, The Affiliated Drum Tower Hospital of Nanjing University Medical School, Zhongshan Road 321, Nanjing, 210008 China; 2 Jiangsu Key Laboratory for Molecular Medicine, Medical School of Nanjing University, 22 Hankou Road, Nanjing, 210093 China

**Keywords:** Stroke, Stroke

## Abstract

Accumulating of evidence suggests that activation of nuclear factor-kappa B (NF-κB) and mitogen-activated protein kinases (MAPKs) exacerbates early brain injury (EBI) following subarachnoid hemorrhage (SAH) by provoking pro-inflammatory and pro-apoptotic signaling. Myeloid differentiation primary response protein 88 (MyD88) is an endogenous adaptor protein in the toll-like receptors (TLRs) and interleukin (IL) -1β family signaling pathways and acts as a bottle neck in the NF-κB and MAPK pathways. Here, we used ST2825, a selective inhibitor of MyD88, to clarify whether inhibiting MyD88 could provide neuroprotection in EBI following SAH. Our results showed that the expression of MyD88 was markedly increased at 24 h post SAH. Intracerebroventricular injection of ST2825 significantly reduced the expression of MyD88 at 24 h post SAH. Involvement of MAPKs and NF-κB signaling pathways was revealed that ST2825 inhibited SAH-induced phosphorylation of TAK1, p38 and JNK, the nuclear translocation of NF-κB p65, and degradation of IκBα. Further, ST2825 administration diminished the SAH-induced inflammatory response and apoptosis. As a result, SAH-induced EBI was alleviated and neurological deficits caused by SAH were reversed. Our findings suggest that MyD88 inhibition confers marked neuroprotection against EBI following SAH. Therefore, MyD88 might be a promising new molecular target for the treatment of SAH.

## Introduction

Subarachnoid hemorrhage (SAH), usually caused by rupturing of intracranial aneurysms, is a life-threatening cerebral vascular disease. SAH mostly affects middle-aged patients with high disability and mortality rates, which, thus, imposes a heavy burden on society and economy^1^. Despite considerable improvements in diagnosis and treatment, the mortality and disability rates of SAH patients remain high. Early brain injury (EBI) and cerebral vasospasm are two major complications that often present in patients suffering from SAH. Past studies have focused primarily on cerebral vasospasm and the reduction of angiographic vasospasm did not translate into a measurable clinical benefit in clinical trials^2,3^. Recent studies have indicated that the pathophysiological event occurring within 72 h post SAH, as termed as EBI, is the most important factor determining the prognosis of patients suffering from SAH^4^.

Convincing data have implicated a role of inflammation and subsequent apoptosis in the development of EBI^5,6^. Inflammatory signaling is up-regulated in both SAH patients and experimental SAH animals, e.g. the expression of toll-like receptors (TLRs), nuclear factor-kappa B (NF-κB), Interleukin (IL) -1β and tumor necrosis factor (TNF) -α were increased in cerebrospinal fluid (CSF), cortex tissue and subarachnoid arteries. NF-κB signaling activation raises SAH-induced inflammatory responses and leads to worse SAH outcomes^7–14^. The severity of early inflammation on admission is linked to poor neurological grades in SAH patients, accompanied by later fever, malaise, leukocytosis, increased blood brain barrier (BBB) permeability, brain edema, small vessel thrombosis and delayed ischemic neurological deficits (DINDs)^15–18^. Although the exact relationship between inflammation and EBI are not totally understood, the result that inhibition of the inflammation process could relieve EBI has been proved in SAH models^19–23^. Additionally, activation of mitogen-activated protein kinases (MAPKs), particularly c-jun-N-terminal kinases (JNK) and p38, could exacerbate EBI by provoking pro-apoptotic and pro-inflammatory cellular signaling^24^. On the other hand, inhibition of p38 and JNK may ameliorate EBI after SAH^25–27^. Thus, NF-κB and MAPK pathways have been considered to be targets therapeutically.

Myeloid differentiation primary response protein 88 (MyD88), an adaptor protein in the TIR and IL-1 family signaling pathways^28^, was originally identified in a myeloid differentiation primary response. It is activated in mouse M1 myeloid precursors following IL-6-induced terminal differentiation^29^. MyD88, as a bottle neck in Toll/IL-l signaling, is composed of an N-terminal death domain and a C-terminal TLR/IL-1R homology domain. Ligand binding to TLR/IL-1R family members results in the association of MyD88 to the cytoplasmic tail of receptors, which initiates the signaling cascade that leads to the activation of NF-κB and MAPK^30^. Activation of both NF-κB and MAPK could provoke pro-apoptotic and pro-inflammatory cellular signaling. Moreover, in a previous study in our laboratory, up-regulation of MyD88 was found early after SAH and lasted at least 7 days^11^. ST2825, a synthetic analogue of MyD88, is a MyD88-specific inhibitor by interfering with MyD88 homodimerization. ST2825 has been applied in different models of human diseases^31,32^. Taking into account all these backgrounds, the purpose of this study was to investigate whether inhibition of MyD88 with its specific inhibitor ST2825 could ameliorate EBI following SAH and analyze the possible molecular mechanism involved.

## Results

### The mortality and general observation are recorded

**Figure 1:**
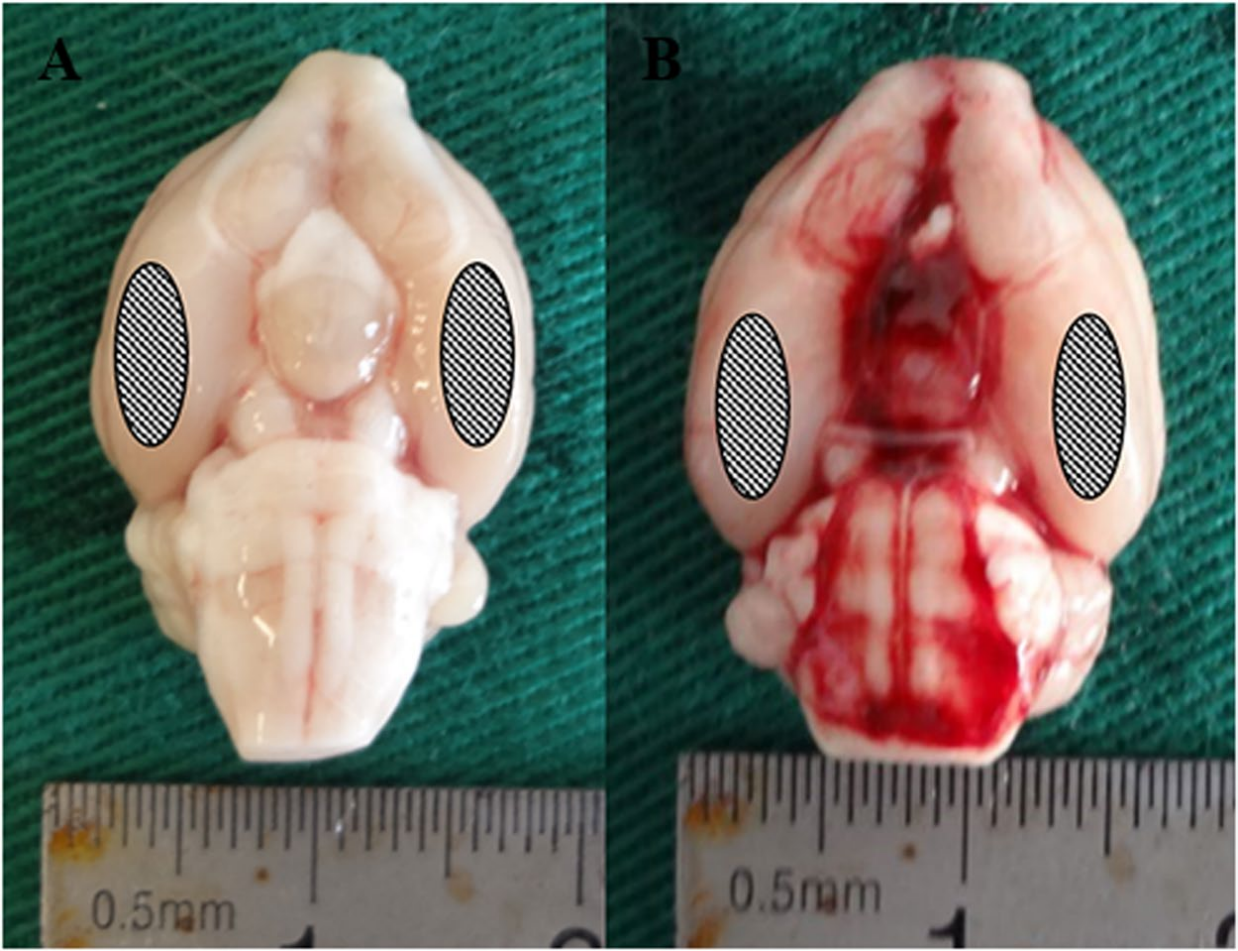
Representative photographs of rat brains after surgery. In the sham-operated rats (**a**), there is no blood clotting throughout the brain. In the SAH group (**b**), the inferior basal temporal lobe was always stained by blood. Hence, the brain tissue adjacent to the clotted blood was taken to the analysis. The site of the obtained sample was indicated with ovals. SAH, subarachnoid hemorrhage.

No significant changes in body temperature or injected arterial blood gas data were detectable in any of the experimental groups. Intracerebroventricular injection of vehicle or ST2825 did not significantly alter arterial blood gas and heart rate in rats. The mortality rates in tow cohorts together were 0% (0/48) in the sham group and 15.5% (36/232) in the SAH rats. The mortality among SAH, vehicle and ST2825 treatment groups was not significantly different (data not shown). Three rats with SAH were excluded later from the study because of little blood in prechiasmatic cistern but lots of blood clot in the frontal lobe instead. The blood clots could easily be found on surface of temporal lobe and around the basilar arteries in SAH rats (Fig. 1b), while there was no visible blood in sham rats (Fig. 1a).

### MyD88 is activated post SAH in rats and ST2825 reduces the levels of MyD88 in SAH rats

**Figure 2:**
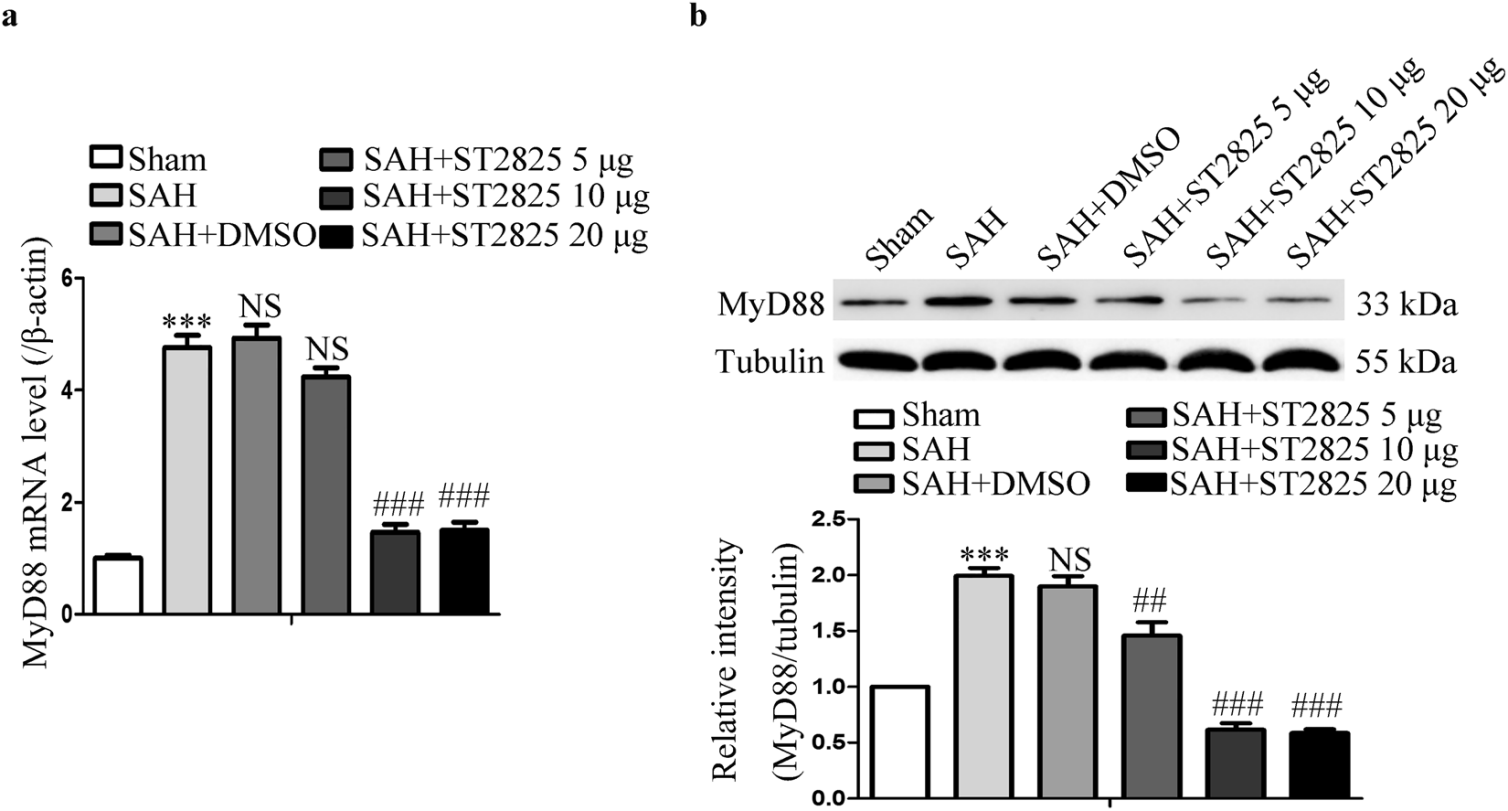
Effect of ST2825 treatment on the expression of MyD88. (**a**) Representative real-time PCR for the expression of MyD88 mRNA in the brain cortex post SAH. (**b**) Representative Western blot to show the level of MyD88 protein. The upper panel shows representative protein levels of MyD88. Tubulin was detected as a loading control. The bottom panel shows quantitative data of MyD88. Data are expressed as mean ± SD (n = 6 in each group). MyD88, Myeloid differentiation primary response protein 88, Definition of SAH is the same as in Fig. 2. ***p < 0.001compared with the sham group, ^##^p < 0.01 compared with the SAH group, ^###^p < 0.001 compared with the SAH group, NS, no statistic difference compared with the SAH group.

To determine whether MyD88 is activated after SAH, tissue extracts from basal temporal lobe were examined for the mRNA and protein levels of MyD88 by real-time PCR and western blotting. The expression of MyD88 increased significantly in both mRNA and protein levels post SAH, which is consistent with the previous study (Fig. 2)^11^. To determine the capability of ST2825 to inhibit MyD88 expression, 5, 10 or 20 μg of ST2825 per rat were ICV administered 30 min post SAH. The result showed that ST2825 administration decreased the expression of MyD88 in a dose-dependent manner. Both 10 μg and 20 μg of ST2825 administration could markedly decrease the expression of MyD88 compared with SAH rats or vehicle-treated SAH rats. Because no severe complications were observed at both middle (10 μg) and high (20 μg) doses tested, these two doses were then chosen to examine its neuroprotective effects on EBI following SAH.

### MyD88 inhibition prevents SAH-induced nuclear translocation of NF-κB p65 and degradation of IκBα protein

**Figure 3:**
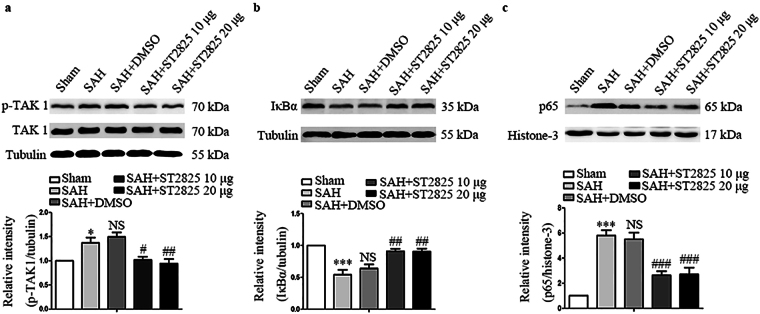
Effects of MyD88 inhibition on p-TAK1, IκBα, and p65 expression 24 h post SAH. Western blots show TAK1, p-TAK1 (**a**), IκBα (**b**) and p65 (**c**) levels following SAH. The upper panel shows representative protein levels of TAK1, p-TAK1, IκBα, and p65. The bottom panels are quantitative data of p-TAK1, IκBα, and p65. Tubulin and histone-3 were used as loading controls. Data are expressed as mean ± SD (n = 6 in each group). p-TAK1, phosphorylated transforming growth factor-β-activated kinase 1, IκBα, inhibitor of NF-κB, Definition of SAH is the same as in Fig. 2. *p < 0.05 compared with the sham group, ***p < 0.001 compared with the sham group, ^#^p < 0.05 compared with the SAH group, ^##^p < 0.01 compared with the SAH group, ^###^p < 0.001 compared with the SAH group, NS, no statistic difference compared with the SAH group.

Activation of MyD88 leads to the activation of TAK1, which, in turn, leads to the activation of NF-κB pathway. To investigate whether inhibition of MyD88 by ST2825 could prevent activation of NF-κB pathway, we evaluated the expression of TAK1, a downstream protein of MyD88, and NF-κB subunits, p65 and IκBα. Upon phosphorylation and subsequent degradation of IκBα, NF-κB activates and translocates to the nucleus, where it binds to DNA and activates the transcription of various genes especially pro-apoptosis and pro-inflammatory genes^33^. In line with the data from a previous study, the level of total TAK1 kept constant after SAH and p-TAK1 increased significantly after SAH, indicating that TAK1 was activated post SAH (Fig. 3a)^34^. While ST2825 treatment significantly reduced the levels of p-TAK1. Our results also revealed significant differences in IκBα and p65 expression among the five groups (Fig. 3b,c). A basal level of IκBα was high in the basal temporal lobe from the rats of the sham group, whereas it was substantially reduced in SAH rats and reversed in ST2825 treatment group, suggesting that ST2825 administration prevented the SAH-induced IκBα-degradation. Then, nuclear fractions were isolated from basal temporal lobe and probed for NF-κB subunit p65 via western blot. The results demonstrated that the level of p65 significantly increased at 24 h after SAH compared to those in the sham group and returned to the basal level after ST2825 treatment.

### MyD88 inhibition downregulates SAH-induced activation of MAPK signaling pathway

**Figure 4:**
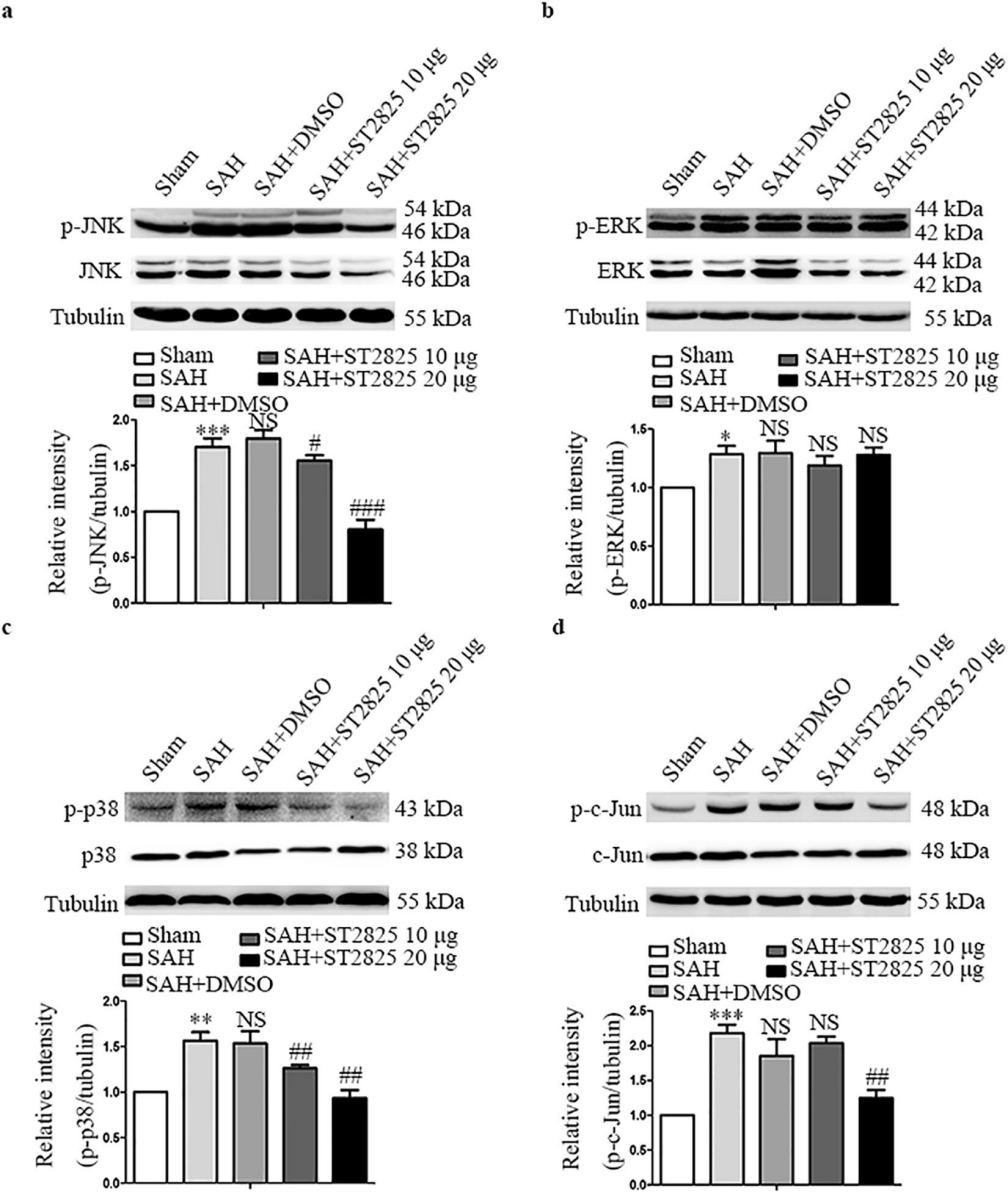
Effect of MyD88 inhibition on the expression of activated JNK, ERK, p38, and c-Jun at 24 h post SAH. Western blots show JNK, p-JNK (**a**), ERK, p-ERK1/2 (**b**), p-38, p-p38 (**c**), and c-Jun, p-c-Jun (**d**) levels following SAH. The upper panel shows representative protein levels of JNK, p-JNK, ERK1/2, p-ERK1/2, p-38, p-p38, c-Jun and p-c-Jun. The bottom panels are quantitative data of p-JNK, p-ERK1/2, p-p38, and p-c-Jun. Tubulin was used as loading control. Data are expressed as mean ± SD (n = 6 in each group). JNK, c-jun N-terminal kinase, ERK, extracellular regulated protein kinases Definition of SAH is the same as in Fig. 2. *p < 0.05 compared with the sham group, **p < 0.01 compared with the sham group, ***p < 0.001 compared with the sham group, ^#^p < 0.05 compared with the SAH group, ^##^p < 0.01 compared with the SAH group, ^###^p < 0.001 compared with the SAH group, NS, no statistic difference compared with the SAH group.

In addition to NF-κB signaling pathway, activation of MyD88 leads to the activation of MAPK pathway, which also plays a key role in the pathogenesis of early brain injury after SAH. To determine the effect of MyD88 inhibition on MAPK signaling in EBI in this study, we investigated the activation of MAPK family such as JNK, ERK1/2 and p38 by western blot. The levels of total JNK, ERK, p38 and c-Jun kept constant after SAH and ST2825 treatment did not affect the levels of total JNK, ERK, p38 and c-Jun after SAH. Our data demonstrated significant differences in p-p38, p-ERK1/2, p-JNK and p-c-Jun expression among the five groups. A significant increase in p-JNK, p-ERK1/2 and p-p38 expression was observed in SAH rats (Fig. 4). Single-dose intracerebroventricular administration of 20 μg of ST2825 prevented the SAH-induced phosphorylation of JNK and p38, but not ERK. Administration of 10 μg of ST2825 failed to prevent the SAH-induced phosphorylation of JNK and p38. C-Jun, a downstream target for phosphorylation by JNK and an important factor in neuronal apoptosis induced by SAH, was evaluated. The result showed that ST2825 treatment reduced the level of the activated c-Jun, confirming the neuroprotective effect of ST2825 after SAH.

### MyD88 inhibition attenuates SAH-induced inflammatory response

**Figure 5:**
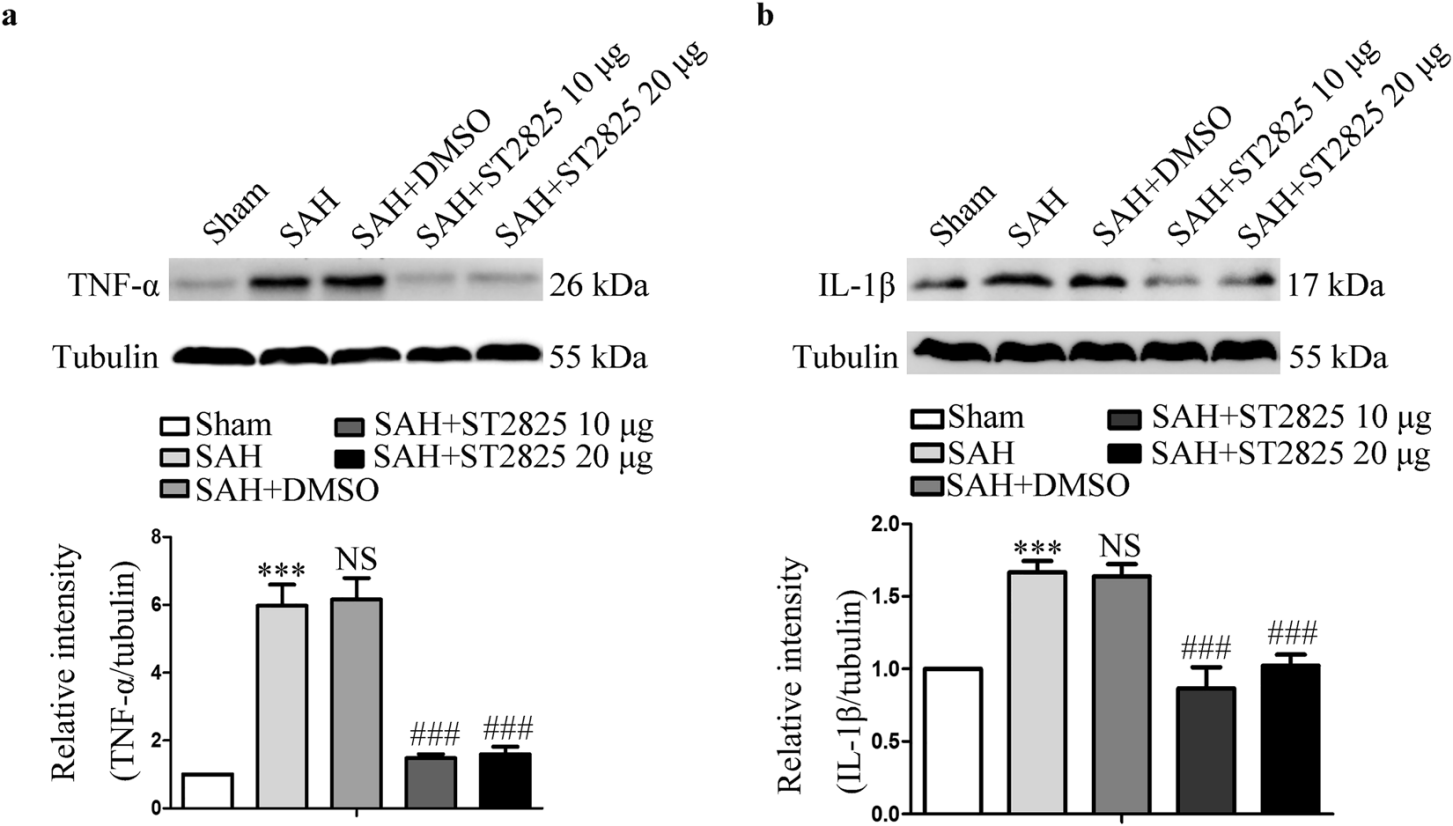
Effects of MyD88 inhibition on TNF-α and IL-1β levels 24 h post-SAH. A substantial increase in TNF-α (**a**) and IL-1β (**b**) was found in anterior basal temporal lobes from SAH rats 24 h after SAH. MyD88 inhibition successfully reduced SAH-induced increase of TNF-α and IL-1β expression. The upper panel shows representative protein levels of TNF-α and IL-1β. The bottom panels are quantitative data. Tubulin was used as loading control. Data are expressed as mean ± SD (n = 6 in each group). TNF-α, tumor necrosis factor-α, IL-1β, Interleukin-1β, Definition of SAH is the same as in Fig. 2. ***p < 0.001 compared with the sham group, ^###^p < 0.001 compared with the SAH group, NS, no statistic difference compared with the SAH group.

Activation of both NF-κB and MAPK signaling pathways could induce pro-inflammatory gene expression leading to the production of pro-inflammatory cytokines such as TNF-α and IL-1β. The previous studies have reported that TNF-α and IL-1β were released in the early stage after SAH and leads to an exacerbation of EBI. To further analyze the effect of MyD88 inhibition on inflammatory response post SAH, the levels of TNF-α and IL-1β were measured^11,21^. Likewise, statistically significant difference was revealed in TNF-α and IL-1β expression among the five groups (Fig. 5). The levels of both TNF-α and IL-1β increased following SAH as has been reported previously^11^. Administration of ST2825 could significantly reduce the synthesis of TNF-α and IL-1β, indicating that inflammatory reaction post SAH was inhibited.

### Neuronal apoptosis is attenuated by MyD88 inhibition

**Figure 6:**
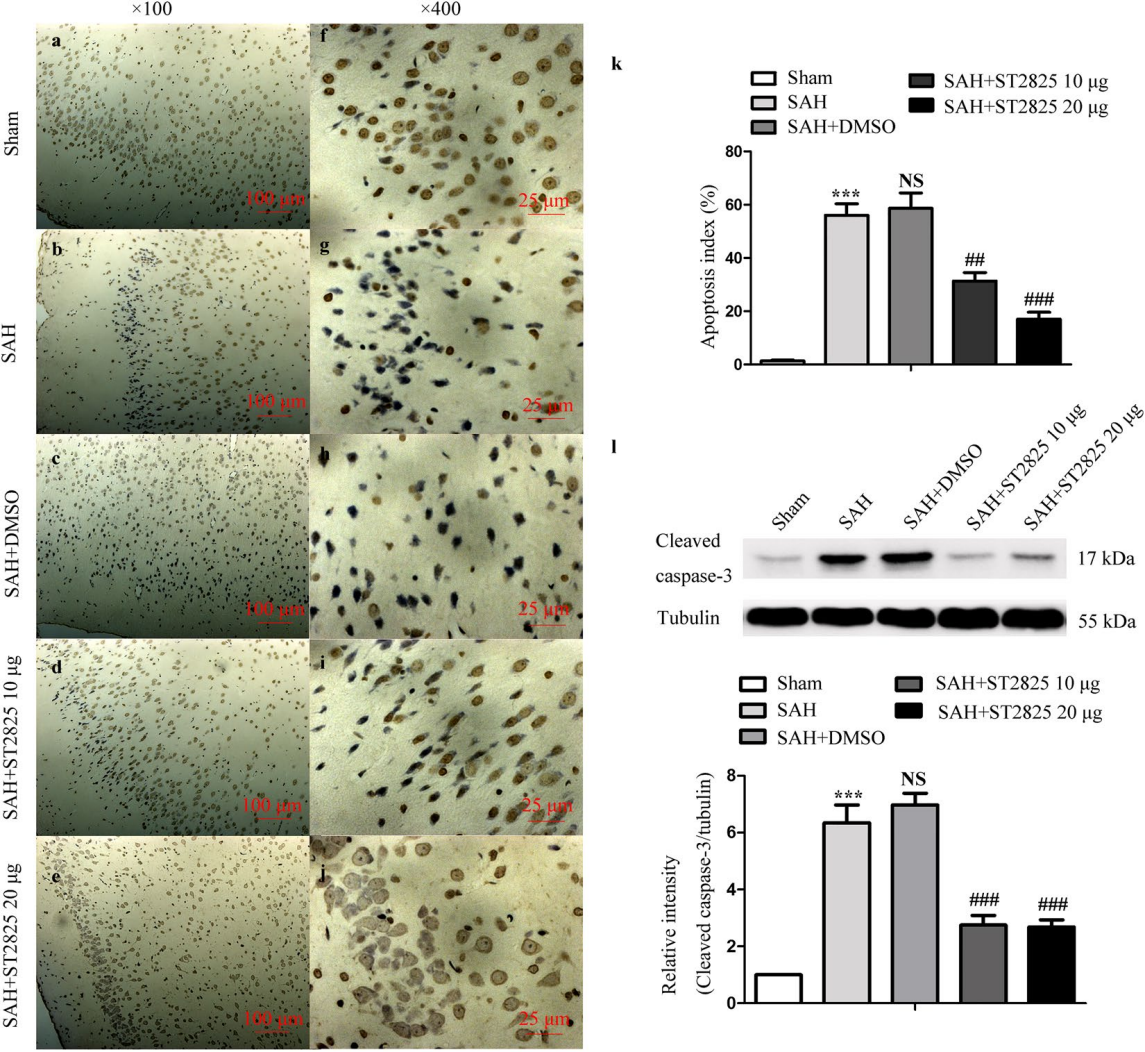
ST2825 treatment inhibited cell apoptosis in the anterior basal temporal lobe at 24 h post-SAH. (**a**–**j**) Representative photomicrographs of TUNEL staining in the inferior basal temporal lobe (**a**–**e**, ×100, **f**–**j**, ×400). (**k**) Statistical data revealed that ST2825 treatment significantly reduced the TUNEL-positive cells compared with SAH and DMSO treated groups. (**l**) MyD88 inhibition reduced the levels of cleaved caspase-3 in the anterior basal temporal lobe at 24 h post SAH. The upper panel shows representative protein levels of cleaved caspase-3. Tubulin was detected as a loading control. The bottom panel shows quantitative data of cleaved caspase-3. Data are expressed as mean ± SD (n = 6 in each group). ***p < 0.001 compared with the sham group, ^##^p < 0.01 compared with the SAH group, ^###^p < 0.001 compared with the SAH group, NS, no statistic difference compared with the SAH group.

MyD88 inhibition was shown to play a neuroprotective effect in animal model of some diseases^35,36^. In this study, SAH-induced activation of NF-κB and MAPK signaling pathways were prevented by administration of ST2825. Activation of NF-κB and MAPK may initiate apoptosis, which is the main pathological process in EBI^37^. Caspase-3 can, in turn, be activated, which is an important indicator of apoptosis. We then analyzed whether the activation of caspase-3 was coupled with the activation of NF-κB and MAPK signaling pathways with Western blotting by evaluating cleaved caspase-3 level^38^. To further analyze the SAH-induced apoptosis and effect of the two drugs, TUNEL assay was designed to detect apoptotic cells that undergo extensive DNA degradation during the late stages of apoptosis with the cortex samples at day 2 after SAH. The results revealed significant difference in apoptosis index and cleaved-caspase-3 expression among the groups. we found a significant increase of cleaved-caspase-3 in SAH and SAH + vehicle groups compared to the sham group (Fig. 6l). Notably, ST2825 treatment significantly decreased the level of cleaved-caspase-3 when compared to the SAH and SAH + vehicle-treated samples. Likewise, SAH increased the number of TUNEL positive cells in the basal temporal lobe at 24 h after SAH (Fig. 6a–k). Inhibition of MyD88 decreased the amount of TUNEL positive cells in SAH rats compared to the untreated or vehicle treated animals. Besides, this result indicated that 20 μg of ST2825 might afford better neuroprotection.

### ST2825 treatment prevents the clinical neurological function and prevents brain tissue from damage after SAH

**Figure 7:**
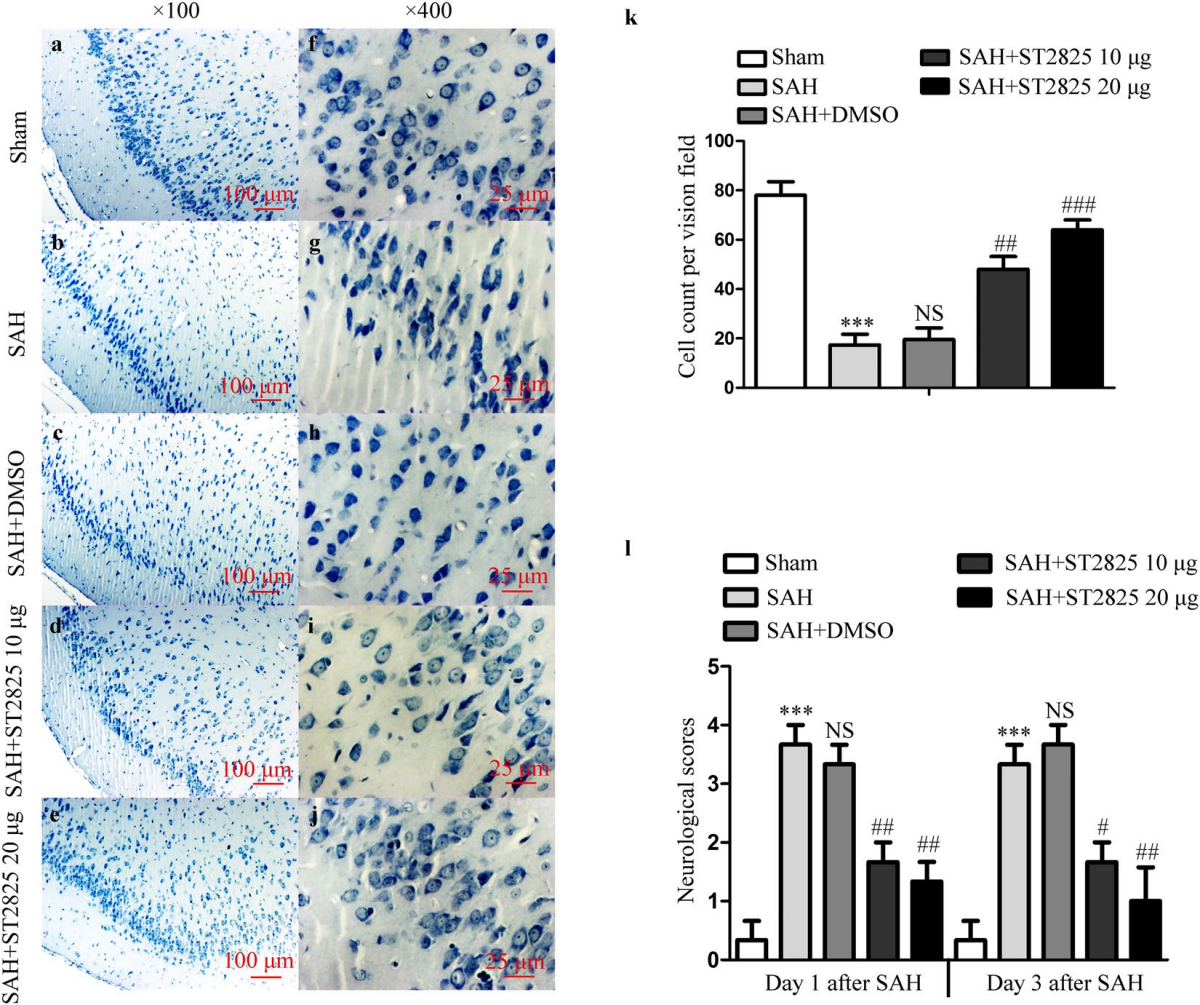
ST2825 treatment ameliorated brain tissue damage and clinical neurological after SAH. (**a**–**j**) Representative slides of Nissl staining at two different magnifications (**a**–**e**, x100, **f**–**j**, ×400) to visualize the neuronal cell outline and structure. SAH reduced the number of the neurons, and treatment of ST2825 preserved neurons from damage, including neuron loss and degeneration. Cells in the SAH and DMSO treated groups were arranged sparsely and the cell outline was fuzzy compared to sham group. (**k**) Cell counts per visual field (×400) was quantified in the slides with Nissl staining. (**l**) Neurological assessment of SAH animals treated with DMSO or ST2825. In comparison with the control group, SAH significantly increased the neurological scores both at 24 h and 72 h post-SAH. ST2825-treated rats exhibited significant improvement in clinical behavioral function at both 24 and 72 h after injury when compared to the SAH rats or DMSO treated rats. Data are expressed as mean ± SD (n = 6 in each group). ***p < 0.001 compared with the sham group, ^#^p < 0.05 compared with the SAH group, ^##^p < 0.01 compared with the SAH group, ^###^p < 0.001 compared with the SAH group, NS, no statistic difference compared with the SAH group.

To identify the neuroprotective effect of ST2825 on behavioral improvement, mean neurological scores were evaluated with a separate cohort of rats. Nonparametric tests revealed significant differences in neurological scores among different groups. SAH induced prominent impairment of the clinical behavioral function at 24 h and 72 h (Fig. 7l). There were no significant differences between SAH and SAH + vehicle-treated rats at both time points. However, ST2825-treated rats exhibited significant improvement in clinical behavioral function at both 24 and 72 h after SAH when compared to the SAH rats or vehicle treated rats. Finally, we stained the brain sections with cresyl violet (Nissl stain) to count the number of intact versus non-intact neurons 72 h post SAH with and without ST2825 treatment. One-way ANOVA analysis showed a significant difference in the number of intact neurons among the four groups (Fig. 7a–k). A large proportion of neurons in the SAH group or vehicle treated group exhibited pyknotic or fragmented nuclei, whereas ST2825 treatment significantly increase the number of intact neurons compared to the SAH group.

## Discussion

The main findings of this study are summarized as follows. First, direct intracerebroventricular delivery of ST2825, a selective MyD88 inhibitor, reduced the expression of MyD88 post SAH. Secondly, NF-κB and MAPK signaling pathways were prevented by administration of ST2825, thus inflammatory response and apoptosis were attenuated. Thirdly, MyD88 inhibition improved the neurologic deficits and prevented brain tissue from damage after SAH.

All TLRs, with the exception of TLR3, recruit MyD88 to their receptor complex, as do members of the IL-1 receptor family. MyD88 interacts with IL-1R associated kinase (IRAK)-4 through its death domain. In turn, IRAK-4 activates other members of the IRAK family, like IRAK-1. This process results in the activation of TRAF6, along with other E2 ubiquitin protein ligases, which activate a complex containing TAK1, TAK1-binding protein 1(TAB1), TAB2, and TAB3. Activation of the TAK1/TAB complex triggers both the MAPK and NF-κB signaling pathways^39^. It has been well demonstrated that ST2825 acts as a potent and selective inhibitor of MyD88. Mechanistically, ST2825 can interfere with recruitment of IRAK1 and IRAK4 by MyD88, causing inhibition of MAPK and NF-κB signaling pathways^35,36^. Our data showed that ST2825 suppress the expression of MyD88. This may due to the fact that synthesis of pro-inflammatory factors such as IL-1β and TNF-α was inhibited by administration of ST2825, thus the IL-1β/MyD88/NF-κB/IL-1β cycle was broken, which in turn down-regulated the expression of MyD88. However, the exact mechanism that ST2825 decreased MyD88 expression is needed to be elucidated in future. As shown in our previous studies, p-TAK1 was activated after SAH and inhibition of TAK1 could notably attenuate SAH-induced brain injury^34^. In the current study, SAH was associated with a significant increase of p-TAK1 expression, ST2825 treatment successfully inhibit the activation of TAK1.

The previous studies have reported that MyD88 is a critical regulator of apoptosis, which is mainly mediated by the downstream pathways NF-κB p65, p38 MAPKK, JNK and ERK-1/2^28,30,39^. Evidences show that the activation of the NF-κB and MAPK pathways contributes to EBI after SAH, and inhibition of these pathways offer neuroprotective effects against SAH^21,27,34^. To identify the molecular mechanisms by which MyD88 inhibition provides neuroprotection after SAH, we first focused on the activation of the NF-κB pathway. we found that inhibition of MyD88 resulted in less NF-κB activation and less neuronal apoptosis *in vivo*. NF-κB has emerged as one of the most promising molecular targets in the prevention of EBI^9,40,41^. NF-κB resides in the inactive state in the cytoplasm as a heterotrimer consisting of p50, p65 and IκBα subunits. An IκBα kinase, IKK, phosphorylates serine residues in IκBα at position. Upon phosphorylation and subsequent degradation of IκBα, NF-κB activates and translocates to the nucleus, where it binds to DNA and activates the transcription of various genes^33^. In this study, we report that SAH was associated with significant IκBα-degradation as well as increased nuclear localization of p65 in the basal temporal lobe at 24 h after SAH. MyD88 inhibition significantly reduced IκBα-degradation as well as NF-κB p65 nuclear translocation. At the same time, inhibition of MyD88 was found to be selectively prevented the SAH-induced phosphorylation of p38 and JNK, but not ERK1/2. Together, the above results demonstrate a selective involvement of MyD88 in the various signaling pathways mobilized in the pathological process of EBI following SAH.

Activation of both NF-κB and MAPKs by over-expression of MyD88 can initiate the transcription of inflammatory cytokines leading to the production of pro-inflammatory cytokines and chemokines and activation of immune cells^30,42^. SAH has long been known to induce an inflammatory response in blood vessels and neuronal tissues. Elevation of TNF-α and IL-1β were reported in the early stage after SAH and leads to an exacerbation of EBI^7–11^. Consistent with the previous work, our data demonstrated that SAH induced a significant increase of TNF-α and IL-1β levels. MyD88 inhibition significantly reduced the release of TNF-α and IL-1β, indicating that SAH induced inflammatory response could be markedly alleviated by MyD88 inhibition.

Collectively, the present study identified MyD88 as a novel upstream mediator of the apoptosis and inflammatory response in EBI following SAH. Selective inhibition of MyD88 by ST2825 reduced the activation of p38, JNK and NF-κB, effectively prevented neuronal apoptosis, inflammatory response, and attenuated neurological deficits in SAH. These data shed new light on the treatment of SAH, and suggest that ST2825 may be an effective drug therapy for EBI after SAH. However, we used only one single-dose intracerbroventricular treatment to evaluate the neuroprotective role of ST2825 in the current study. Whether systemic injection of ST2825 provided similar beneficial effects remains to be further investigated.

## Methods

### Animal Preparation

All procedures were approved by the Animal Care and Use Committee of Nanjing University and were conformed to Guide for the Care and Use of Laboratory Animals by National Institutes of Health. Male Sprague-Dawley (SD) rats (6–8 weeks, 300 to 330 g) were obtained from Animal Center of Jinling hospital (Nanjing, China). The rats were housed in a humidity controlled room (25 ± 1 °C, 12 h light/dark cycle) and were raised with free access to water and food.

### Prechiasmatic Cistern Blood Injection for SAH Model

Experimental SAH models were performed as reported previously^38,43^. The rats were anesthetized with chloral hydrate (0.4 mg/kg, IP, Jinling hospital). The hair on the head and near the inguinal region was carefully shaved and then the rats were positioned prone in a stereotactic frame. After disinfection, a midline scalp incision was made and a 1 mm hole was drilled 8.0 mm anterior to the bregma in the midline of the skull, through the skull bone, down to duramater without perforating the underlying matter. Then the animals were positioned supination. After disinfection again we use insulin syringe (1 ml 29 G × 1/2 m, 0.33 mm × 12.7 mm) (BD Bioscience, San Jose, CA) to draw 300 ml volume of blood from femoral artery. The needle was advanced 11 mm into the prechiasmatic cistern through the burr hole, at a 45° angle to the vertical plane, and the 300 ml blood was injected into the prechiasmatic cistern over 20 seconds. Loss of cerebrospinal fluid (CSF) and bleeding from the midline vessels were prevented by plugging the burr hole with bone wax prior to inserting the needle. Sham animals were injected with 300 ml normal saline. After injection, animals were kept in a 30 °C, heads-down position for 20 minutes. Arterial blood samples were analyzed intermittently to maintain pO2, pCO2, and pH, parameters within normal physiological ranges. To maintain fluid balance, all rats were supplemented with 2 ml of 0.9% NaCl administered subcutaneously. After recovery from anesthesia, the rats were returned to their cages and housed at 25 ± 1 °C. Rats that died during surgery or during surgical recovery were excluded, and the procedure was repeated until final group sizes reached the planned experimental number.

### Experimental Design

ST2825 was purchased from MedChem Express (Monmouth Junction, NJ) and freshly prepared in dimethyl sulfoxide (DMSO) just before ICV injection. In our dose response study, we tested different dosages at 5, 10 and 20 μg per rat. ST2825 solution or DMSO (5 μl) was injected into the left lateral ventricle 15 min post SAH using a 10 μl Hamilton microsyringe. Coordinates for the injection placement were 1.0 mm posterior to bregma, 1.4 mm lateral to midline, and 4.4 mm below the skull surface and the injection duration was 10 min^44^. Following the behavioral test, brain tissue was harvested for biochemical and histopathological analysis at 24 h post SAH.

Total 240 rats were randomly equally divided into the sham (surgery with normal saline insult), SAH, SAH + DMSO (SAH treated with vehicle DMSO 5 μl), SAH + ST2825 5 μg (SAH treated with 5 μg of ST2825 in DMSO, the same preparation below), SAH + ST2825 10 μg, and SAH + ST2825 20 μg groups (40 rats/group). Of them, eight rats with SAH were excluded later from the study because of little blood in prechiasmatic cistern but lots of blood clot in the frontal lobe instead, and twenty-nine (SAH, n = 6; SAH + DMSO, n = 7; SAH + ST2825 5 μg, n = 6; SAH + ST2825 10 μg, n = 4; SAH + ST2825 20 μg, n = 6) SAH rats died before sacrifice.

A separate group of rats was followed for 72 h functional testing after which brain tissue was collected for histological assessment. We used 40 animals in this study, assigned to following groups: sham (n = 8); SAH (n = 8); SAH + DMSO (n = 8); SAH + ST2825 10 μg (n = 8) and SAH + ST2825 20 μg (n = 8). Two rats with SAH were excluded because of blood clot in the frontal lobe. Seven (SAH, n = 2; SAH + DMSO, n = 2; SAH + ST2825 10 μg, n = 2; SAH + ST2825 20 μg, n = 1) SAH rats died before the intended sacrificed.

### Clinical Evaluation

Three behavioral activity examinations were performed at day 1 and day 3 after SAH using the previously described scoring system modified for rodent subjects^44^. Scoring was conducted by two independent researchers blinded to the groups. Grading of neurologic deficits was as follows, 1, no neurologic deficit (scores = 0); 2, minimum or suspicious neurologic deficit (scores = 1); 3, mild neurologic deficit (scores = 2–3); 4, severe neurologic deficit (scores = 4–6).

### Perfusion–fixation and Tissue Preparation

Animals were anesthetized as above, and perfused through the left cardiac ventricle with normal saline (4 °C) until effluent from the right atrium was clear. Animals which had obvious clots in the prechiasmatic cistern were selected to further analyze. The temporal lobe tissue (Fig. 1) which was near the hematoma was harvested on ice after blood clots on the tissue cleared carefully. The tissue was stored in −80 °C till further use for biochemical analysis. For histological examination, the rats were perfused with normal saline (4 °C) followed by 4% buffered paraformaldehyde (4 °C) and then the brains were immersed in 4% buffered paraformaldehyde (4 °C) for further study.

### RNA isolation and quantitative real-time PCR

Rat brain tissue was isolated using TRIzol Reagent (TaKaRa Biotechnology, Tokyo, Japan) as manufacturer’s instructions. The concentration and quantity of the RNA were determined by spectrophotometric analysis at 260 and 280 nm. The isolated RNA was reverse-transcribed to cDNA using Reverse Transcriptase Reagent (TaKaRa, Biotechnology) and oligodT primers. Quantitative real-time PCR analysis was performed using the Agilent Technologies Strata gene Mx3000 P real-time PCR system (Gene times Technology, Inc.), applying real-time SYBR Green PCR technology. The reaction mixtures contained 1 μl cDNA, 12.5 μl SYBR Green (TaKaRa Biotechnology), 1 μl of each forward and reverse primer (10 μM) and nuclease-free water to a final volume of 25 μl. The primers were synthesized by Life Technologies (Invitrogen, Shanghai, China) and the sequences used were from a data base at NCBI for rat MyD88 and β-actin. The forward and reverse primers for *MyD*88 were 5′-TT-CTCCAACGCTGTCCTGTC-3′ and 5′-AACTGAGATGTGTGCC- CAGG-3′; for β-actin were 5′-AGGGAAATCGTGCGTGAC-3′ and 5′-CGCTCATTGCCGA-TAGTG-3′. After 95 °C for 30 s, 40 PCR cycles were performed, each consisting of a denaturation step (95 °C, 5 s) and an annealing step (60 °C, 30 s). Total RNA concentrations from each sample were normalized by quantity of β-actin messenger RNA (mRNA), and the expression levels of target genes were evaluated by using the 2^−ΔΔCq^ method. All samples were analyzed in triplicate.

### Western Blot Analysis

The whole cell protein extraction and nuclear fractions were prepared as previously described^34^. Forty μg of total protein were loaded in each lane of SDS-PAGE, electrophoresed, and transferred to a nitrocellulose membrane (NC, PALL). The blot containing the transferred protein was blocked in blocking buffer (1 × Tris-buffered saline with Tween 20 with 5% w/v nonfat dry milk without antibody) for 1 h at room temperature followed by overnight primary antibody incubation at 4 °C. The primary antibody was against MyD88 (cat# ab2064), ERK1/2 (cat# ab17942), interleukin (IL)-1β (cat# ab9722), phospho-TGF β-activated kinase- 1(p-TAK1, Ser439, cat# ab109404) (1:1,000 dilution, Abcam, Cambridge, MA), cleaved-caspase-3 (cat# 9661), p-ERK1/2 (cat# 4730 P), p-JNK (cat# 4668 P), p-c-Jun (cat# D47G9), p-P38 (cat# 4511), histone 3 (cat# 9715) (1:1,000 dilution, Cell Signaling Technology, Danvers, MA), β-Tubulin (cat# T6199) (1:1,000 dilution, Sigma-Aldrich, St. Louis, MO), TAK1 (cat# sc-7162), p38 (cat# sc-7149), p65 (cat# sc-372), JNK (cat# SP600125), c-Jun (cat# sc-166540), IκBα (cat# sc-371) and tumour necrosis factor (TNF)-α (cat# sc-52746) (1:200 dilution, Santa Cruz Biotechnology, Santa Cruz, CA). After incubation with secondary antibodies and washing again, the blotted protein bands were visualized by enhanced chemiluminescence Western blot detection reagents (Millipore Corporation, Billerica, MA). Relative changes in protein expression were estimated from the mean pixel density using UN-SCAN-IT, normalized to β-tubulin (H3 for nuclear protein), and calculated as target protein expression/β-tubulin (histone 3) expression ratios.

### Terminal Deoxynucleotidyl Transferase (TdT) dUTP Nick End Labeling (TUNEL) Staining

Brain tissues fixed with 4% paraformaldehyde were dipped in 20% saccharose PBS for 2 days and then in 30% saccharose PBS for another 2 days to remove water in the tissues. Sections 7 μm in thickness were sliced and washed with PBS. TUNEL assay was conducted using an *In Situ* Cell Death Detection Kit (Roche Inc., Indianapolis, USA) just as the given protocol. The extent of brain damage was evaluated by the apoptotic index, defined as the average percentage of TUNEL-positive cells in each section counted in 10 cortical microscopic fields (at 400x magnification). Five sections spaced a minimum of 100 μm apart were obtained from each animal and used for quantification. The final average percentage of apoptotic index of the five sections was regarded as the data for each sample.

### Nissl Staining and Cell Counting

For Nissl staining, the 4 μm sections were hydrated in 1% toluidine blue at 50 °C for 20 minutes. After rinsing with double distilled water, the sections were dehydrated and mounted with permount. Normal neurons have relatively big cell body, rich in cytoplasm, with one or two big round nuclei, while damaged cells show shrunken cell bodies, condensed nuclei, dark cytoplasm, and many empty vesicles. Cell counting was restricted to the temporal lobe. Six random high-power fields (400×) in each coronary section were chosen, and the mean number of surviving neurons in the six views was regarded as the data of each section. A total of four sections from each animal were used for quantification. The final average number of the four sections was regarded as the data for each sample. Data were presented as the number of neurons per high-power field. All the processes were conducted by two pathologists blinded to the grouping.

### Statistical Analysis

All data were presented as mean ± SD. SPSS 17.0 was used for statistical analysis of the data. All data were subjected to one-way ANOVA. Differences between experimental groups were determined by the Fisher’s LSD post-test. Statistical significance was inferred at P < 0.05.
