# Author Correction: Inhibition of myeloid differentiation primary response protein 88 provides neuroprotection in early brain injury following experimental subarachnoid haemorrhage

**DOI:** 10.1038/s41598-024-58400-4

**Published:** 2024-04-05

**Authors:** Huiying Yan, Dingding Zhang, Yongxiang Wei, Hongbin Ni, Weibang Liang, Huasheng Zhang, Shuangying Hao, Wei Jin, Kuanyu Li, Chun-Hua Hang

**Affiliations:** 1 Department of Neurosurgery, The Affiliated Drum Tower Hospital of Nanjing University Medical School, Zhongshan Road 321, Nanjing, 210008 China; 2 Jiangsu Key Laboratory for Molecular Medicine, Medical School of Nanjing University, 22 Hankou Road, Nanjing, 210093 China

Correction to: *Scientific Reports* 10.1038/s41598-017-16124-8, published online 17 November 2017

The Article contains errors. Due to errors during figure assembly, the sample “Sham” in Figure 6a shows an image belonging to the dataset “SAH + ST2825 10 µg” and therefore partially overlaps with Figure 6d. An updated Figure 6 with the correct data for the sample “Sham” appears below as Figure 1:

**Figure 1:**
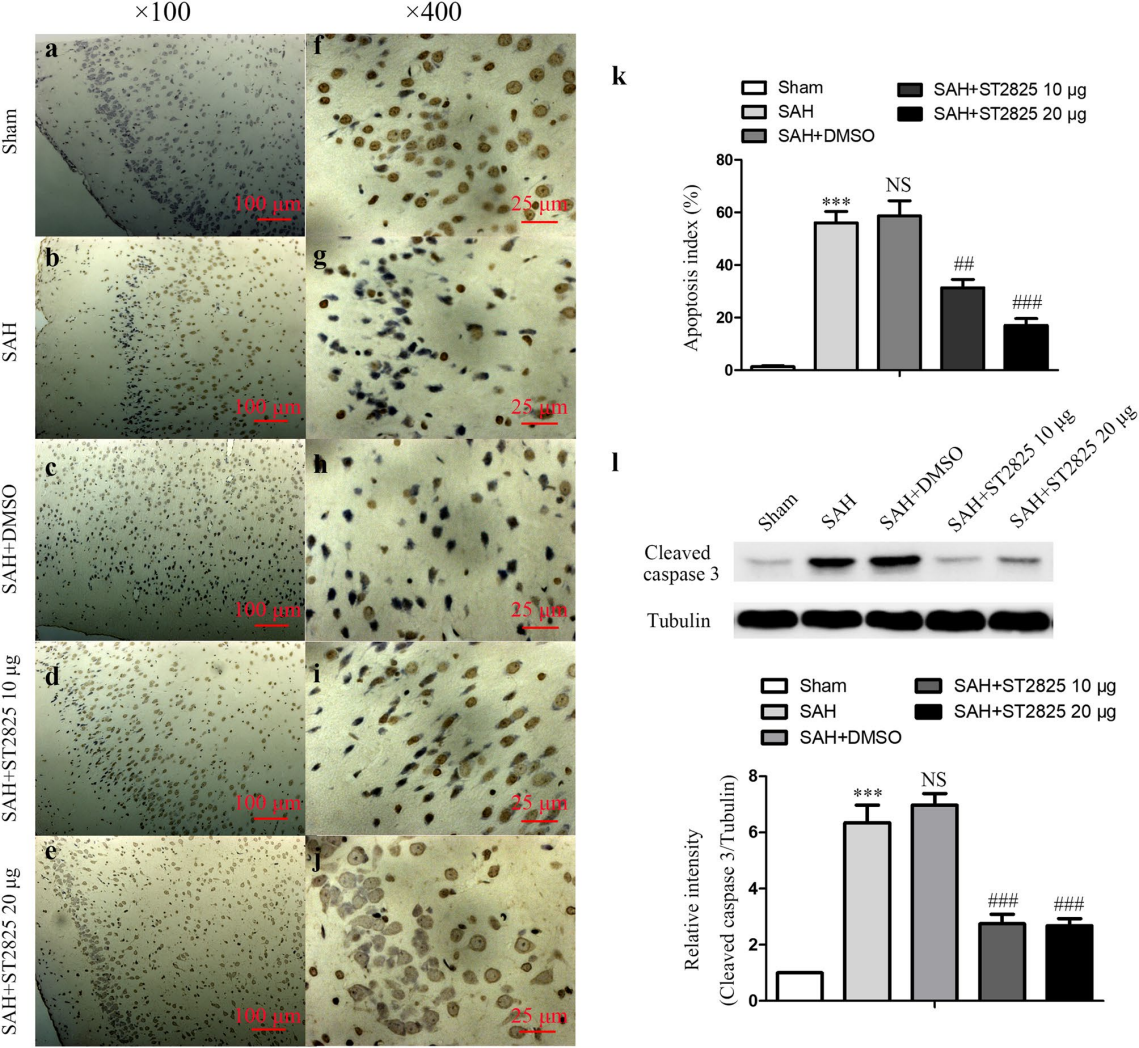
ST2825 treatment inhibited cell apoptosis in the anterior basal temporal lobe at 24 h post-SAH. (**a**–**j**) Representative photomicrographs of TUNEL staining in the inferior basal temporal lobe (**a**–**e**, × 100, **f**–**j**, × 400). (**k**) Statistical data revealed that ST2825 treatment significantly reduced the TUNEL-positive cells compared with SAH and DMSO treated groups. (**l**) MyD88 inhibition reduced the levels of cleaved caspase-3 in the anterior basal temporal lobe at 24 h post SAH. The upper panel shows representative protein levels of cleaved caspase-3. Tubulin was detected as a loading control. The bottom panel shows quantitative data of cleaved caspase-3. Data are expressed as mean ± SD (n = 6 in each group). ***p < 0.001 compared with the sham group, ^##^*p* < 0.01 compared with the SAH group, ^###^*p* < 0.001 compared with the SAH group, NS, no statistic difference compared with the SAH group.

In addition, in Figure 7b the sample shown for “SAH” belongs to the dataset “SAH + DMSO” and therefore partially overlaps with Figure 7c. An updated Figure 7 with the corrected sample for “SAH” appears below as Figure 2:

**Figure 2:**
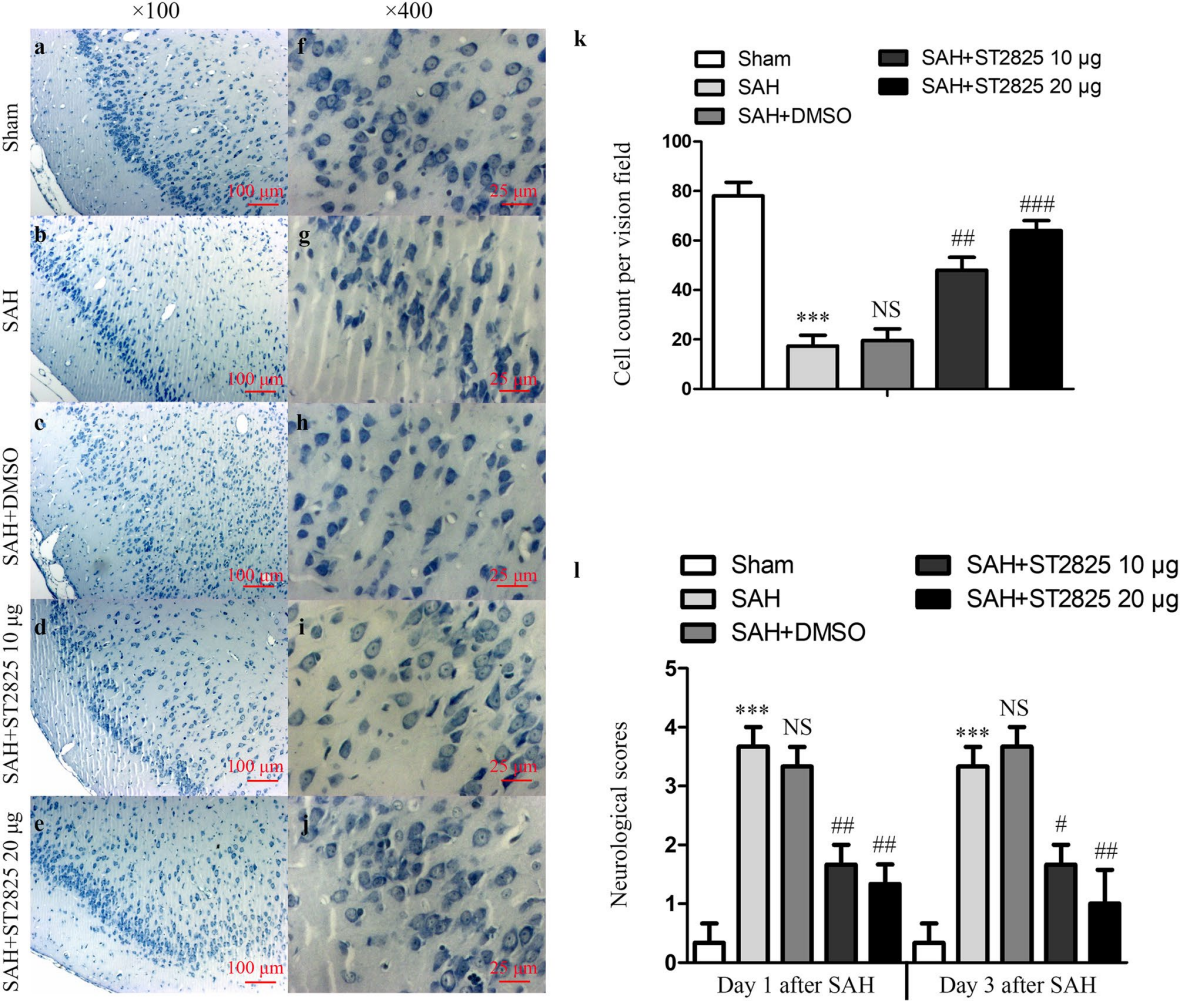
ST2825 treatment ameliorated brain tissue damage and clinical neurological after SAH. (**a**–**j**) Representative slides of Nissl staining at two different magnifications (**a**–**e**, × 100, **f**–**j**, × 400) to visualize the neuronal cell outline and structure. SAH reduced the number of the neurons, and treatment of ST2825 preserved neurons from damage, including neuron loss and degeneration. Cells in the SAH and DMSO treated groups were arranged sparsely and the cell outline was fuzzy compared to sham group. (**k**) Cell counts per visual field (× 400) was quantified in the slides with Nissl staining. (**l**) Neurological assessment of SAH animals treated with DMSO or ST2825. In comparison with the control group, SAH significantly increased the neurological scores both at 24 h and 72 h post-SAH. ST2825-treated rats exhibited significant improvement in clinical behavioral function at both 24 and 72 h after injury when compared to the SAH rats or DMSO treated rats. Data are expressed as mean ± SD (n = 6 in each group). ***p < 0.001 compared with the sham group, ^#^*p* < 0.05 compared with the SAH group, ^##^*p* < 0.01 compared with the SAH group, ^###^*p* < 0.001 compared with the SAH group, NS, no statistic difference compared with the SAH group.

